# Wishes and needs of community-dwelling older persons concerning general practice: A qualitative study

**DOI:** 10.1371/journal.pone.0200614

**Published:** 2018-07-17

**Authors:** Sophie C. E. van Blijswijk, Claudia S. de Waard, Petra G. van Peet, Dagmar Keizer, Margaret von Faber, Margot W. M. de Waal, Wendy P. J. den Elzen, Jacobijn Gussekloo, Jeanet W. Blom

**Affiliations:** 1 Department of Public Health and Primary Care, Leiden University Medical Center, Leiden, The Netherlands; 2 University of Applied Sciences, Leiden, The Netherlands; 3 Department of Clinical Chemistry and Laboratory Medicine, Leiden University Medical Center, Leiden, The Netherlands; 4 Department of Internal Medicine, Section Gerontology and Geriatrics, Leiden University Medical Center, Leiden, The Netherlands; Texas Technical University Health Sciences Center, UNITED STATES

## Abstract

**Introduction:**

Community-dwelling older persons often experience hindering health complaints that disturb daily activities. If general practitioners (GPs) are unaware of such complaints, this could lead to a mismatch in provided care and needed or expected care. In this qualitative study with community-dwelling older persons we investigated how older persons experience hindering health complaints, how they deal with them, and what they expect from their GP.

**Methods:**

Participants (aged ≥80 years) with pain and/or problems with walking/standing were invited to participate in a (group)interview about hindering health problems and expectations from general practice. Data were analysed using the framework method and results were discussed with a client panel.

**Results:**

Participants experienced various hindering health complaints in addition to pain and/or problems with walking/standing. Complaints affecting social activities were experienced as the most hindering. Participants actively tried to remain independent and, to achieve this, GPs were expected to be involved and be easily accessible. However, they did not expect specific help from their GP for pain or problems with walking/standing. Suggestions for improvement of care from GPs included optimisation of accessibility (continuous availability by telephone), a yearly check including medication review, open communication, and empathy and support during major life events.

**Conclusions:**

According to older persons with hindering health complaints, GPs could improve their accessibility/relationship with patients by: 1) continuous telephonic availability, 2) initiating regular contact with medication reviews, and 3) initiating support during major life events. This might lower the reported barriers to contact the GP for hindering health complaints.

## Introduction

The general practitioner (GP) is the primary medical professional for all community-dwelling older persons in the Netherlands. GPs serve as the first contact for health problems and often have a long-term relationship with their patients. They are also the gatekeepers for secondary medical care [1]. In many general practices, the GP collaborates with other primary care professionals (e.g. GP assistants and practice nurses) who are often involved in care for patients with chronic illnesses, and older patients.

GPs are expected to provide proactive care for community-dwelling older persons: the aim is to identify (a high risk of) problems in an early stage and prevent worsening, enabling older persons to live longer at home [2]. To adequately and effectively deliver this type of proactive care, GPs need to be aware of the complaints/limitations experienced by their patients. However, a striking difference has been observed between the percentage of older persons reporting one or both of the most frequent reported hindering complaints (i.e. pain and/or problems with walking/standing), and the percentage of patients with a registration of these complaints in the GP’s electronic patient registration [3]. This difference could lead to mismatch between provided care and needed, expected or wished for care.

Therefore, this difference was investigated with the aim to improve health care provided by GPs. For this, we performed a qualitative study in which we explored 1) the hindrance that community-dwelling older persons experience in daily life due to their health complaints, 2) their own initiatives to handle this hindrance, and 3) what they expect from the general practice when they experience these complaints and hindrance.

## Methods

### Study design and population

This qualitative study is embedded in the follow-up of the Integrated Systematic Care for Older People (ISCOPE) study, a large healthcare innovation trial. Details on the ISCOPE study are already published [4]: briefly, in ISCOPE, all eligible patients aged ≥75 years in 59 Dutch general practices (n = 11,476) were invited to participate (September 2009–2010). They received the postal ISCOPE questionnaire (response rate 63.5%) with questions on four health domains [5]. A selection of the participants (37.2%, n = 2,713) was visited at home at baseline and after 12 months (80.8%, n = 2,192) to collect more information on their health status [4].

In 2015, all previously participating practices were asked to participate in a follow-up study. Participants who were visited after 12 months and who were still eligible to participate received the ISCOPE questionnaires and a new informed consent form, including a question about participation in a group interview related to their wishes and needs from general practice. Assistance was offered to complete the questionnaires. Next, participants who reported problems with walking and/or standing or pain in the open-ended question in ISCOPE questionnaire [3] were invited to participate to ensure that they could recognise each other’s complaints to facilitate discussion. Potential participants were contacted by telephone to schedule an appointment. The GPs were not informed about the participation of their patients. Interviews were treated confidentially and all participants provided informed consent on audio record. The Medical Ethical committee of the Leiden University Medical Center approved the study.

### Focus groups and individual interviews

Group interviews were preferred because interactive discussion between older persons with different experiences and ideas leads to additional information [6, 7]. To allow participation of older persons with functional limitations (i.e. sensory impairment and/or problems with mobility), we organised small focus groups (maximum of five participants) in various Dutch cities and offered transport by taxi. In addition, for participants with mobility problems, home visits were planned for individual interviews.

The research plan, interview guide (S1 File) and a draft of a list of codes were based on literature (Health Belief Model [8–10], Andersen-Newman model [11, 12], Theoretical Domains Framework [13, 14], and other studies [15–19]), a meeting with the research team, a meeting with the Regional Elderly Advisory Board and two test interviews which were held with members of this Board. The Regional Elderly Advisory Board is a client panel of 15 older persons (aged 60–90 years) who reflect on research proposals/results and healthcare policy in the Leiden area. A summary of the present study is shown in the supporting information files (S2 File).

One researcher moderated the semi-structured focus groups (PGP or CSW) and the individual interviews (CSW, SCEB or DK) which took (on average) two hours each. Another researcher (CSW, SCEB or DK) made field notes. After each (group) interview, field notes were discussed and data saturation was checked. If needed, changes to the interview guide were made.

### Data analysis

Data were analysed thematically (qualitative content analyses) using the Framework Method. This method allows for a deductive, inductive or combined approach depending on the research question. The data is coded and summarized in a matrix to facilitate comparison of the data within and across (group) interviews. An essential part of the analysis is the iterative discussion between the researchers on the assigned codes and the relevance of the emerging themes [20]. All (group) interviews were recorded and transcribed verbatim. The developed list of codes was inductively adapted. For three (group) interviews, within-case matrices were composed independently by two researchers (CSW, SCEB or DK) and discussed. For two other (group) interviews, the within-case matrix was composed by one researcher (CSW or SCEB) and discussed with the other researcher. Data from the within-case matrices were combined in a cross-case matrix. Data from all other (group) interviews were summarized and compared with the cross-case matrix. The findings were discussed with the research team and a summary of findings was discussed with the Regional Elderly Advisory Board. Recommendations were based upon suggestions from participants themselves and derived from their wishes and expectations. Data were processed and analysed with Speech Exec pro transcrib and Atlas.ti 6.2.

## Results

First, the characteristics of the study participants are presented, including their experienced health complaints and limitations and how they manage these. Second, we present their expectations regarding their GP and the general practice. Expectations and recommendations (based on participants’ suggestions, and meetings with the authors and the Regional Elderly Advisory Board in which the wishes and expectations of the participants were discussed) are summarized in Table 1.

**Table 1:** Results and recommendations per theme.

Summary of findings				Recommendations
*I Patient characteristics*: *Health complaints and impact*				
A diverse range of complaints was experienced and often accepted. Social limitations were considered worst. GPs were not expected to solve all problems.			Ask pro-actively about (social) limitations.	
*I Patient characteristics*: *Self-management of health complaints and limitations*				
Continuing activities were tried despite limitations. Accepting dependency and asking for help was difficult. Informal and professional care was appreciated.		Ask pro-actively whether help is needed to be able to continue their activities.		
*II Expectations of their GP*: *Expectations of treatment*				
Not much was expected of GPs for most complaints (including pain and problems standing/walking) due to acceptance and low expectations of treatment.	Focus more on coping with limitations instead of curing diseases.			
*II Expectations of their GP*: *Shared decision-making*				
To live at home as long as possible, some guidance was expected regarding diagnostics, therapy, and to welfare organisations.				Guide patients to further diagnostics, therapy and welfare organisations if needed.
*II Expectations of their GP*: *Pro-active care*				
Some considered contact with their GP to be their own responsibility. Others indicated that they are afraid to lose sight of the ‘big picture’ of their health due to age and limitations and would therefore like the GP to take more initiative.				Discuss with patients whether they need a more pro-active attitude from the general practice because they lose sight of the ‘big picture’ of their health situation.
*II Expectations of their GP*: *Attentive care (i*.*e*. *support and empathy)*				
For a good patient-doctor relationship it was considered important to also recognise social and emotional matters.				Be attentive: especially around major life events, as well as for previously mentioned complaints, even if these cannot be cured.
*II Expectations of their GP*: *Attainability and accessibility*				
Some participants felt their complaints do not warrant bothering the GP, and were even more reluctant to contact their GP after a negative experience or for a known but unsolved problem.				Establish a yearly moment of contact initiated by the general practice. Offer help to older patients and promote the services of the practice nurse to all older patients
Telephone accessibility during office hours was too limited. The emergency option was not suitable because older persons are reluctant to use this option.				Continuous telephone accessibility during office hours.
Some participants did not know why the assistant (instead of the GP) asks about their complaints and gives advice on health complaints.				Be clear why the GP’s assistant asks clarifying questions in order to make an appointment with the GP.
*II Expectations of their GP*: *Coordinating health care and medication*				
Unsure whether their medication was up-to-date and an apparent lack of communication with medical specialists was perceived.				Follow-up on information from medical specialists and perform a yearly review of medications and communicate this to the patient, even if no changes are made.

### I. Characteristics of study participants

**Fig 1:**
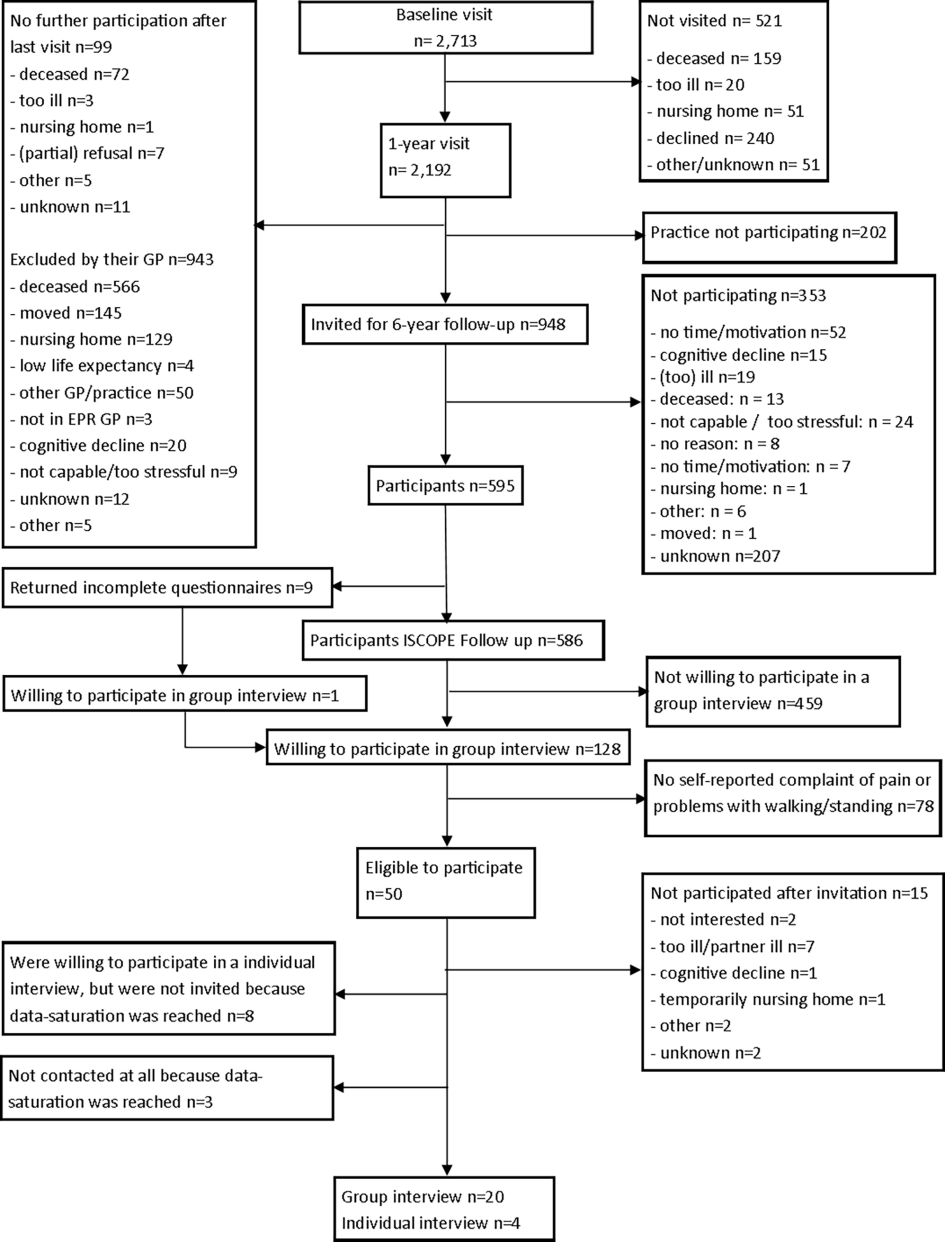
Flowchart of participants.

Of the 948 older persons invited for the follow-up of the ISCOPE study, 595 (62.8%) returned a questionnaire, of which 128 (21.5%) were willing to participate in the present study. Seventy-eight older persons did not meet the inclusion criteria since they did not self-report pain or problems with walking/standing in the ISCOPE questionnaire. Of the 50 potential participants, 15 declined participation for various reasons (e.g. illness, illness of their spouse, or no longer interested), and 11 persons were not contacted because data saturation was reached. Finally, 24 persons (from 20 different general practices) participated in a (group) interview (Fig 1).

The median age of the 24 participants was 85.7 (IQR 83.5–90.5) years and 18 were female. Most participants lived independently with others (n = 8) or alone (n = 14), and two lived in a home for the elderly. Of the 24 participants, 14 were widowed. Multimorbidity (n = 19) and polypharmacy (n = 17) were common (Table 2, S3 File).

**Table 2:** Baseline characteristics of participants (n = 24).

		Total	Focus groups	Individual
				interview
		(n = 24)	(n = 20)	(n = 4)
Characteristic		n	n	n
Age in years (median, IQR)		85.7 (83.5; 90.5)	86.1 (83.5; 90.5)	85.2 (82.3; 93.3)
Female		18	15	3
Marital status				
	Married	7	6	1
	Divorced	2	2	0
	Widowed	14	11	3
	Living agreement/not married	1	1	0
Living situation				
	Independent, alone	14	11	3
	Independent, with others	8	7	1
	Home for the elderly	2	2	0
Multi-morbidity (>1 chronic disease)		19	16	3
Polypharmacy (>3 medications)		17	14	3
Self-reported problems				
	Pain	16	14	2
	Problems with walking and/or standing	19	16	3
	Additional other problems	15	13	2

#### Health complaints and impact

Although selection of these participants was based on the presence of pain and/or problems with walking/standing, the complaints that participants experienced were not limited to these two. Fifteen participants experienced a wide range of additional complaints and limitations, including incontinence, loneliness, and visual and hearing problems, which were often recognised by other participants. Participants indicated that they experienced social limitations (e.g. feeling alone, being less involved in society) and health complaints causing social limitations (e.g. losing the capacity to drive, not being able to eat with cutlery), or less socially accepted complaints (e.g. faecal incontinence) as the most bothersome. Almost all participants were both aware and slightly anxious of possible future problems, especially cognitive problems.

Without exception, they considered most of their health complaints to be age related. In combination with acknowledgment of the ‘good life’ that they had already lived, this often led to the acceptance of their health problems; however, the impact on their vitality, daily life and (social) activities was more difficult to accept. For example, an 83-year-old female stated:

“I think to myself: yes woman, you just have to accept it. You’re 83 years old, it’s normal deterioration and he [the GP] can’t give you new bones or a new arm. You’re 83 years old, you’re blessed and fortunate. Well—what else do you want?”

#### Self-management of health complaints and limitations

The participants agreed that it was important to ‘keep moving’, both literally and figuratively, e.g. keeping up-to-date with new (technical) developments. This was considered to be their own responsibility. However, due to their limitations they lacked the flexibility to adjust to various changes, as this 85-year-old female explains:

“Well, I happen to have a computer and an Ipad and all that stuff, because I need it for my volunteer work, but I’m not very good at it. (…) It changes too quickly for me because—just when I’m used to doing it one way—it has to be done differently.”

Thus, some participants stopped participating in activities because of these difficulties, or because they no longer wanted to do them. Others forced themselves to participate in (social) activities because they acknowledged the positive influence on their mood, social network and physical health. As this 96-year-old female states:

“On the one hand, I dislike shopping for groceries, but—on the other hand—at least it keeps you a little bit more active and that’s good.”

In general, most older persons tried to maintain their independence and stay in control of their own situation as long as possible, e.g. by using aiding devices. Some took specific precautions, such as moving to a smaller home or applying for a home for the elderly or nursing home. Most participants were grateful for the informal care received from their children, and the availability and high standard of professional home care; nevertheless, some drawbacks (e.g. many different homecare workers) were also mentioned.

The risk of struggling too long before accepting help from others was also acknowledged. Almost all participants agreed that they find it hard to admit to themselves and others that they need help, as this 91-year-old female explains:

“It’s difficult to be dependent. But—at a certain point—you have to accept it. My kids always say: don’t nag mam, you’ve always taken care of us, now we’re taking care of you.”

### II. Expectations of their GP concerning their health complaints

Important themes that were discussed were expectations of treatment, shared decision-making, pro-active care, attentive care (i.e. support and empathy), attainability and accessibility and coordination of health care and medication (Table 1).

#### Expectations of treatment

In general, participants were satisfied with their GP. They considered their GP to be skilled and trustworthy. Participants who had experienced a lack of communication in general, or after a major life event, were less satisfied. Expectations of the benefits of treatment were low due to old age and to the lack of effect of previous treatments. This applied to most complaints, including pain and problems with walking/standing for which acceptance was high and belief in improvement was low. Since participants did not expect their GP to solve all their problems they did not always discuss their complaints either. Another reason not to discuss their complaints was ‘feeling ashamed’ of their complaints. Some participants wished that their GP would focus more on coping with limitations instead of curing their complaints.

#### Shared decision-making

Most participants confirmed they would like to live independently in their own home as long as reasonably possible. In an effort to make the limitations caused by their health problems manageable, they expected guidance from their GP towards adequate diagnostics, referral for a second opinion, (non-)medical therapy, aiding devices, and/or social care services. They relied on their GP to guide them through medical possibilities (taking the risks and possible benefits into account) but tended to make their own choices concerning interventions, as this 86-year old female stated:

“I’ve always expected a little more guidance. I mean—like directions for where to go for a helping hand”

#### Pro-active care

Most participants would like the GP to ‘keep an eye on them’ because they felt they had lost a sense of mastery; e.g. they felt they had lost sight of the ‘big picture’ of their health now that they were getting older and experiencing more limitations. They felt more vulnerable than they used to be and felt that a pro-active attitude from the GP was needed to prevent further health problems. They appreciated home visits if needed, but wondered why this was seldom initiated by the GP. This is explained by these 90-year-old and 81-year-old ladies:

“Well, it would be comforting to me (…) if they monitored me more often. (…) Especially when you’re at an age that you can’t…. when you’re getting older.”

“I’d like them to take the initiative to invite me for a check-up on some things, to get some lab tests done. She told me I should do that once a year, but she doesn’t remind me—so two years later I think…”

Participants felt that introducing a yearly moment of contact initiated by the general practice could help to make them feel at ease and lower the barrier for them to contact the general practice. It was not necessary for the GP to visit them personally, a contact with the practice nurse or a GP’s assistant by telephone or during a consultation would also be appreciated. During this conversation, they could discuss known complaints, laboratory test results and the situation at home. Participants who already had regular contact with the GP or practice nurse because of chronic diseases or polypharmacy, appreciated this and did not need additional attention. This 81-year-old female said this about a yearly appointment:

“It [receiving a yearly invitation for an appointment with the GP] would put me at ease, because I think you’d receive all the test results during that appointment as well.”

In contrast, some participants felt it was their own choice and responsibility to contact the GP if they felt the need to do so. This 86-year-old male stated:

“If tomorrow I were to feel something I didn’t trust or I couldn’t handle, then I would just go see the GP. The GP shouldn’t have to follow-up on me, should he?”

Many participants stated that it was the patient’s responsibility to be well prepared for a doctor’s appointment (i.e. bring a list of questions, a clear request for help, and even suggestions for treatment). They felt capable of doing this (e.g. felt a sense of mastery) and were disappointed when the GP did not use their suggestions in (shared) decisions about further treatment.

#### Attentive care: Support and empathy

It was considered important to have a good patient-doctor relationship based on respect. For some participants this includes not only adequate medical treatment, but also attention for the consequences of a complaint in their (daily) life, their (social) situation, and life events. Follow-up and recognition of their problems was important to them, even without available treatment or when a problem was already solved, as this 88-year-old female pointed out:

“Once I had an infection in my wrist and that was solved—but he’s never asked about it again. That’s a little bit of response you would like to receive, that you feel that we’ve solved the problem together.”

Participants also appreciated receiving attention during negative life events such as the death of a partner, or hospital admission. Some participants were disappointed about this, and this had a considerable impact on the relationship with their GP.

#### Attainability and accessibility

Many participants had the perception that their GP was always busy. Some participants tried to contact their GP as little as possible because they prefer not to bother him with the ‘minor complaints of an older person’, although they sometimes would appreciate advice about these complaints, as this 92-year old female does:

“Well, it would be nice if the GP would visit his patients. I would like that. I’m also dizzy now and again—then I have to support myself by holding on to something. But I’m not going to call the GP for this. No, that’s not something you call your GP for—but it would be nice if he would check up on his patients.”

They were even less inclined to contact their GP when they had a negative experience with their GP (e.g. not apologising for a mistake, or not being available after a life event). They relied on their GP to contact them when new treatments were available, since they expected their GP to be aware of the complaints they had previously mentioned. Some participants stated that they would not dare to ask a second time for help for a known complaint for which no satisfying solution had been found, as this 92-year-old female says:

“Well, I believe it would be good if I discussed my leg problems again, but if the GP doesn’t mention it well… I don’t dare to start the conversation.”

Some participants found it difficult that some GPs worked part-time and were not available all week; others appreciated the larger general practices with several primary healthcare professionals. Not all participants were satisfied with replacement of the regular general practices with GP centres after business hours; however, most understood the need for a separate service, as this 83-year old female explains:

“I used to have a GP who came to check up on a child at 11.00 PM. That’s not happening anymore and that’s something I can understand.

The participants expected their general practice to be easily available by telephone during office hours, and not only during certain time frames. They would like to have the option to discuss with a professional whether an experienced complaint needs to be assessed immediately or not. The separate emergency telephone number (or emergency option on the answering machine) available in most practices was not helpful because most participants hesitated to use this. There was no interest in online communication with the GP. This 83-year-old male said:

“You can’t expect all GPs to be continuously available any more, like they used to be in small villages—but I agree that it’s wrong that you can’t reach your GP because they’re having a lunch break. That shouldn’t be allowed.”

Some participants stated that they had a good relationship with the GP’s assistant. However, the assistant is not always appreciated because some participants felt that the assistant is trying to ‘shield’ the GP from them. Most participants prefer to talk directly to the GP about their complaints, as is clear from these statements from an 83-year-old and a 90-year-old female:

“I feel the assistant protects the GP’s office and the GP like a lion.”

“I sometimes think it’s a drawback that the GP’s assistants start asking what your problems are and what they can tell the GP”

Although appointments with the practice nurse are mostly intended to follow-up on chronic diseases, participants who had appointments with the practice nurse often discussed other (minor) health problems, as well as psychological and social problems. Some participants suggested there should be extra contact with the practice nurse to discuss their health. They felt this could avoid a consultation with the GP, or reveal previously unknown complaints that need follow-up. Participants who had experience with the practice nurse, such as this 81-year-old female, appreciated these contacts and the available time:

“I do visit the practice nurse for diabetes and I feel that this is very helpful. She measures my blood pressure and everything, and discusses my yearly check-up. I have great confidence in that (…) She also asks questions about mental issues, about my husband and everything. So actually she’s really important”

#### Coordinating health care and medication

Participants found it important that their GP played a key role in their health care. GPs were expected to be well informed about their health, to link new knowledge to their health situation if applicable, to communicate regularly with medical specialists, and to have contact with welfare organisations. Some participants had the idea that the GP was not aware of the treatment received from their medical specialist.

Some participants had the experience that, on referral to another physician, some of the medication they used proved to be no longer necessary. Participants were amazed that this could happen and felt that this could be solved with a yearly check of their medication by the GP, if necessary in combination with additional diagnostics. Communication about this, even if no changes are necessary, should be done by the GP since he is responsible for the medication. Information by the assistant or pharmacist is often not appreciated. This 88-year-old female says:

“I take half a tablet because of high blood pressure, but first I had one tablet. When I ran out, I went to the pharmacy and they told me I should take half a tablet from now on. They told me the GP was going to call me about this, but that never happened (…) I think it’s strange that I should hear this from the pharmacy.”

## Discussion

### Summary

The aim of the present study was to formulate recommendations to improve health care from general practice for community-dwelling older persons with self-reported limiting complaints (pain and problems with walking/standing). For this, we investigated their hindering complaints, their initiatives taken to handle this, and their expectations from general practice. However, it became clear that older persons had very few expectations from their GP concerning these complaints, mainly due to their acceptance of these complaints and the perception that these complaints are age-related and not curable. Participants experienced a range of other health complaints and limitations. Complaints affecting social activities were experienced as the most bothersome. In general, older persons were actively trying to remain independent. For this, they expected their GP to be involved in their situation and easily accessible. Shared decision-making, pro-active care, and attentive care (i.e. support and empathy) were considered to be important in their relationship with their GP. Participants felt that the GP was often busy and that accessibility could be improved. They expected the GP to be aware of their complete and up-to-date health status, including a yearly check-up; however, this was not always experienced.

### Strengths and limitations

Strengths of this study are the diverse range of community-dwelling older participants (e.g. in respect of sex, marital status, living situation, multimorbidity and polypharmacy) who were included from a population-based study. All participants experienced pain and/or problems with standing/walking and a wide range of additional complaints and limitations. We captured a broad range of opinions from this diverse population due to the interaction of the participants during focus groups, and the recognition of each other’s complaints. Letters received afterwards (in which participants reflect on the group interviews) confirm this. Involvement of the Regional Elderly Advisory Board helped to further focus our thoughts.

In general, a possible limitation of focus groups is that more sensitive items are not discussed. However, as seen before, this is not necessarily true [6] and our participants spoke openly about items as incontinence and mistakes made by their GP. 21% of the participants of the ISCOPE follow-up agreed to participate in a (group)interview, they are diverse in several important characteristics. Because of his diversity we are confident that we’ve captured a broad range of opinions. We do recommend to further explore the recommendations emerging from this study since this might provide additional insights on the perspectives of specific groups of older persons, e.g. with more severe limitations.

### Comparison with literature

Our study builds on results from a qualitative study among residents of elderly care homes, community-dwelling older persons, and their GPs and coordinating nurses [21]. Our results confirm the importance of autonomy and independence for older persons and the organisational barriers experienced (i.e. triage by the assistants). In both studies, the participants were satisfied with the medical expertise of their GPs, but had some comments about the overall care and communication. In addition, we found that what participants expect from their GP changes due to experienced limitations affecting a sense of mastery: when older persons feel they are ‘losing grip’ and are lacking sight of the ‘big picture’ of their health, they would like their GP to be more attentive and initiate contact on a regular basis. These findings confirm results from our earlier study on the perspectives of older persons screened positive for depressive symptoms, on the causes and solutions for their depressive symptoms [22]. Similarly, we showed that most older persons do not expect professionals to solve all of their problems, but do express a need for support and empathy concerning their hindering complaints. Problems with accessibility by telephone and a preference for one full-time GP have been reported earlier [18]. However, although we also found a preference for continuity of care, it was also found that most participants understood the need for different GP services after business hours and were often satisfied with other employees in their general practice, such as the practice nurse.

### Implications for general practice

It is important for older persons to maintain independence and to be able to live at home as long as possible. It seems that older persons want their GP to take more initiative when they are losing a sense of mastery. Since the experienced sense of mastery can change over time, we recommend that the GP discusses with older patients whether they expect a more pro-active approach from the general practice. Furthermore, GPs could explain to their older patients that it is important that they discuss complaints that limit their daily life with the GP, even if they feel that their complaints are age-related. Instruction of older patients on the role of the assistant and the emergency services in the general practice might be important to ensure that patients not hesitate to inform their GP on new hindering complaints. Adequate communication on these topics between GPs and older patients might lower the barrier for older patients to discuss their health problems with the GP. Good telephone accessibility, the possibility of asking for a longer consult, additional contact hours with the practice nurse, and yearly contact initiated by the general practice, might reduce the feeling some older persons have of a GP who is always busy and difficult to contact. It is important to communicate new developments (e.g. the GP introducing the practice nurse to the patient and informing the patient about a performed medication review) to the patients. Last but not least, it is important for older persons to receive attentive care: care with attention for their personal situation, especially around major life events. Implementing these suggestions in general practice could enhance older persons’ confidence in the GP, and lower the barrier for older persons to contact the general practice.

## Supporting information

S1 File Interview guide (focus groups and individual interviews). (PDF)

S2 File Flowchart of the study. (PDF)

S3 File Characteristics of participants per (group)interview. (PDF)
