# Supplementary material for: Wishes and needs of community-dwelling older persons concerning general practice: A qualitative study
Source: PLoS One. 2018 Jul 17;13(7):e0200614. doi: 10.1371/journal.pone.0200614 (PMC6049904; doi:10.1371/journal.pone.0200614)
Supplement: S1 File — (PDF) [file pone.0200614.s001.pdf]

S1 File. Interview guide (focus groups and individual interviews)

Topics	Timetable	Cumulative
1. Introduction<br>a. Introduction researchers<br>b. Recording the interview<br>c. Confidentiality<br>d. Aim of interview<br>e. Conversationrules<br>f. Duration and break	10 min	10 min
2. Introduction of participants<br>a. Who are you? Where do you live?<br>b. What comes to your mind at first on this topic?<br>c. Vitality of yourself and other older persons	10 min	20 min
3. Mapping hindering complaints<br>a. Which complaint(s) hinder you from day to day?<br>b. Why are these complaints important for you?<br>c. What is their impact on your daily activities?<br>NB. Focus on pain and problems with standing/walking	10 min	30 min
4. Care needs and wishes<br>a. What care needs and wishes do you have concerning these hindering health complaints?<br>b. Who can/should help with these needs and wishes?<br>c. What do you consider important concerning these persons and/or organisations?	25 min	55 min
COFFEEBREAK	15 min	70 min
5. Role of the GP and general practice<br>a. Why did you or did you not visit your GP/general practice with these hindering health complaints?<br>b. What do you expect from your GP/general practice concerning these complaints?<br>NB. Focus on pain and problems with standing/walking	40 min	110 min
6. To conclude<br>a. Thank participants<br>b. Explanation of what will happen with the audiofiles<br>NB. Give present, fill in forms for travel expenses supply parking tickets. Help participant to own transport or taxi.	10 min	120 min
