# Supplementary figures and images for: Wishes and needs of community-dwelling older persons concerning general practice: A qualitative study
Source: PLoS One. 2018 Jul 17;13(7):e0200614. doi: 10.1371/journal.pone.0200614 (PMC6049904; doi:10.1371/journal.pone.0200614)

## S2. Flowchart of the study

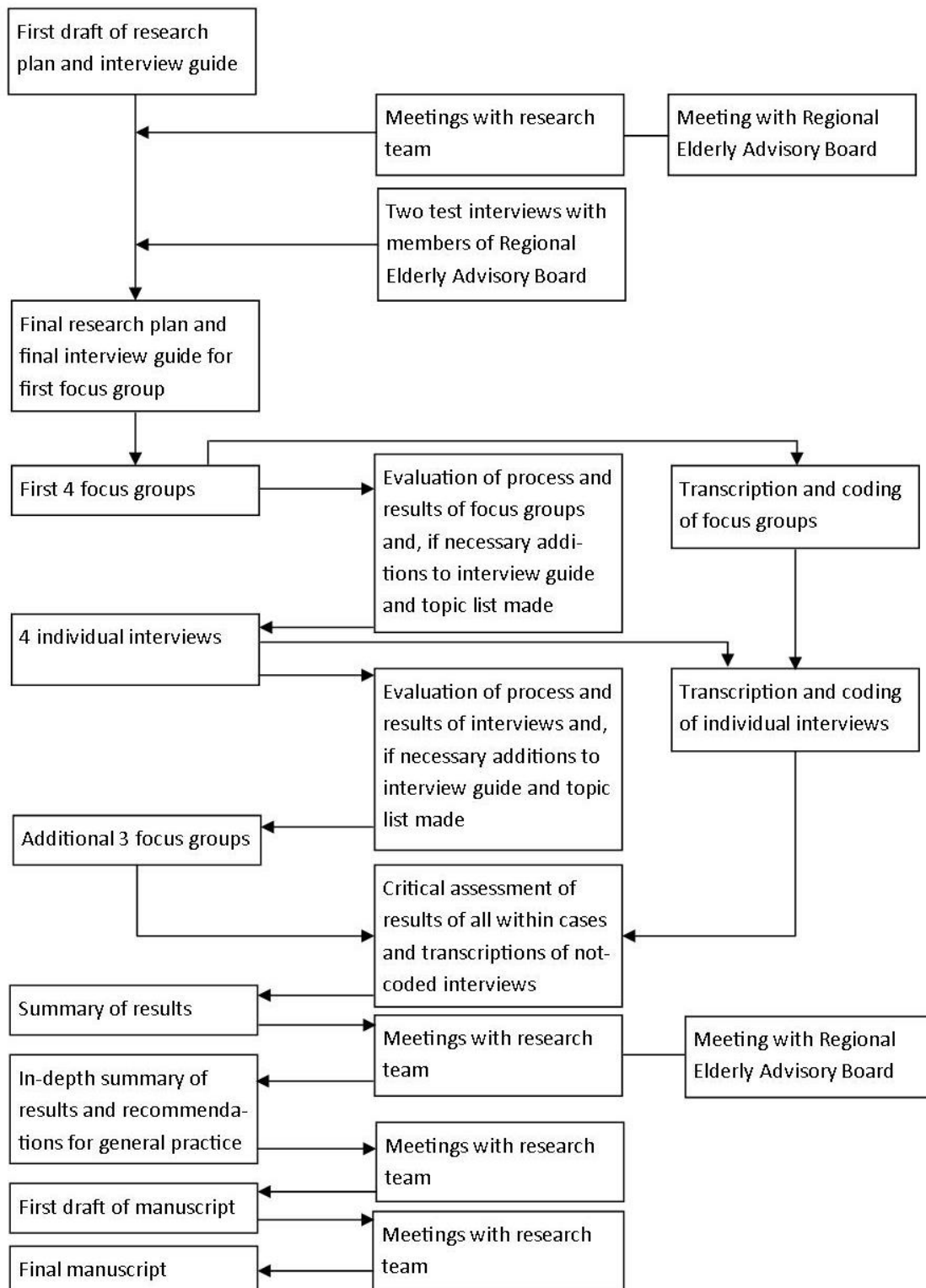

Supplement: S2 File — (PDF) [file pone.0200614.s002.pdf]
