# Supplementary material for: Wishes and needs of community-dwelling older persons concerning general practice: A qualitative study
Source: PLoS One. 2018 Jul 17;13(7):e0200614. doi: 10.1371/journal.pone.0200614 (PMC6049904; doi:10.1371/journal.pone.0200614)
Supplement: S3 File — (PDF) [file pone.0200614.s003.pdf]

S3. Characteristics of participants per (group)interview

Participant	Age	Sex	Marital status	Living situation	Multimorbidity	Polyfarmacy	ISCOPE score	Pain	Problems with walking/standing	Other complaints
1.1	83.1	F	Maried	Independent, with others	Yes	Yes	3	No	Yes	Yes
1.2	86.3	M	Maried	Independent, with others	Yes	Yes	3	Yes	No	No
1.3	86.4	M	Maried	Independent, with others	No	No	0	No	Yes	Yes
2.4	85.8	F	Divorced	Home for the elderly	Yes	Yes	4	Yes	Yes	Yes
2.5	91.1	F	Widowed	Independent, alone	Yes	Yes	3	Yes	Yes	Yes
3.6	81.7	F	Widowed	Independent, alone	Yes	Yes	0	Yes	No	Yes
3.7	81.2	F	Maried	Independent, with others	Yes	Yes	1	Yes	Yes	Yes
3.8	87.9	F	Widowed	Independent, alone	No	No	1	Yes	Yes	No
4.9	92.5	F	Widowed	Independent, alone	Yes	Yes	4	No	Yes	Yes
4.10	82.8	F	Maried	Independent, with others	Yes	Yes	4	Yes	Yes	Yes
4.11	90.0	F	Widowed	Independent, alone	Yes	Yes	3	Yes	Yes	Yes
5.12	98.7	F	Widowed	Independent, alone	No	No	0	No	Yes	No
5.13	92.2	F	Divorced	Home for the elderly	Yes	No	4	Yes	Yes	No
6.14	83.8	M	Living together	Independent, with others	Yes	Yes	2	Yes	Yes	Yes
6.15	83.6	M	Widowed	Independent, alone	Yes	Yes	3	No	Yes	Yes
6.16	89.4	M	Widowed	Independent, alone	Yes	Yes	4	No	Yes	No
6.17	90.6	F	Maried	Independent, with others	No	No	4	Yes	Yes	No
6.18	83.7	F	Widowed	Independent, alone	Yes	Yes	3	Yes	No	Yes
7.19	83.5	F	Widowed	Independent, alone	Yes	No	2	Yes	No	No
7.20	85.2	F	Widowed	Independent, alone	Yes	Yes	4	Yes	Yes	Yes
8.21	85.7	M	Widowed	Independent, alone	No	Yes	2	No	Yes	No
9.22	84.8	F	Widowed	Independent, alone	Yes	No	4	Yes	Yes	Yes
10.23	81.5	F	Maried	Independent, with others	Yes	Yes	2	No	Yes	No
11.24	95.8	F	Widowed	Independent, alone	Yes	Yes	3	Yes	No	Yes
